# Effects of TSA, NaB, Aza in *Lactuca sativa L*. protoplasts and effect of TSA in *Nicotiana benthamiana* protoplasts on cell division and callus formation

**DOI:** 10.1371/journal.pone.0279627

**Published:** 2023-02-24

**Authors:** Seung Hee Choi, Woo Seok Ahn, Myoung Hui Lee, Da Mon Jin, Areum Lee, Eun Yee Jie, Su Ji Ju, Sung Ju Ahn, Suk Weon Kim

**Affiliations:** 1 Biological Resource Center, Korea Research Institute of Bioscience and Biotechnology (KRIBB), Jeongeup, Republic of Korea; 2 Department of Biotechnology, Chonnam National University, Gwangju, Republic of Korea; 3 National Institute of Crop Science, RDA, Wanju, Republic of Korea; 4 Sunchang Research Institute of Health and Longevity, Sunchang, Republic of Korea; 5 Department of Applied Plant Science, Chonnam National University, Gwangju, Republic of Korea; Lovely Professional University, INDIA

## Abstract

Whole-plant regeneration via plant tissue culture is a complex process regulated by several genetic and environmental conditions in plant cell cultures. Recently, epigenetic regulation has been reported to play an important role in plant cell differentiation and establishment of pluripotency. Herein, we tested the effects of chemicals, which interfere with epigenetic regulation, on the plant regeneration from mesophyll protoplasts of lettuce. The used chemicals were histone deacetylase inhibitors trichostatin A (TSA) and sodium butyrate (NaB), and the DNA methyltransferase inhibitor azacytidine (Aza). All three chemicals increased cell division, micro-callus formation and callus proliferation in lettuce protoplasts. Cell division increased by more than 20% with an optimal treatment of the three chemicals. In addition, substantial increase in the callus proliferation rates was observed. In addition, TSA enhances cell division and adventitious shoot formation in the protoplast culture of *Nicotiana benthamiana*. The regenerated tobacco plants from TSA-treated protoplasts did not show morphological changes similar to the control. TSA increased histone H3 acetylation levels and affected the expression of *CDK*, *CYCD3-1*, and *WUS* in tobacco protoplasts. Thus, we investigated the effect of TSA, NaB, and Aza on *Lactuca sativa L*. protoplasts and the effect of TSA on cell division and callus formation in *Nicotiana benthamiana* protoplasts, which facilitates plant regeneration from mesophyll protoplasts. Furthermore, these chemicals can be directly applied as media additives for efficient plant regeneration and crop improvement in various plant species.

## Introduction

Plant regeneration is an important process to repair the loss or injury of plant parts because they are sessile organisms. To date, plant tissue culture studies have established an *in vitro* plant regeneration system for various plants by utilizing the totipotency of plants [1, 2]. Whole-plants regeneration from protoplast cultures involves a series of steps, including protoplast isolation, formation of protoplast-derived callus, shoot regeneration, and root regeneration [3]. These tissue culture steps are controlled by various mechanisms, including gene regulatory networks [4]. To regulate gene regulatory networks, an understanding of the modifications in chromatin structure by DNA methylation and histone modification is very important [5, 6]. Despite having the same genetic background, covalent modifications in chromatin allow cells to express distinct and different characteristics [7]. When chromatin, called heterochromatin, is tightly packed through DNA methylation and/or histone modifications, the genes present in that region have reduced access to the transcriptional machinery, resulting in silenced or decreased expression. However, when chromatin, called euchromatin, is lightly packed, the genes present in that region have increased access to transcriptional machinery, resulting in their active transcription. The main function of histone modifications including histone acetylation, methylation, ubiquitination, phosphorylation, and SUMOylation, is transcriptional regulation [8]. In particular, histone acetylation and deacetylation are mediated by histone acetyltransferases (HATs) and histone deacetylases (HDACs), respectively, to regulate gene expression [9]. DNA methylation, a biological process in which methyl groups are added to adenine and cytosine by DNA methyltransferases, plays an important role in development, aging, genomic imprinting, and repression of transposable elements via gene regulation [10].

Trichostatin A (TSA) is a typical HDAC inhibitor that can bind to HDACs by inserting a long aliphatic chain into the active site of the enzyme, resulting in inhibition of its activity [11]. Sodium butyrate (NaB), a short-chain fatty acid, is another HDAC inhibitor [12]. TSA and other HDAC inhibitors have been used in studies aimed at increasing plant regeneration efficiency. TSA facilitates totipotency in the male gametophyte of *Brassica napus* [13]. TSA and suberoylanilide hydroxamic acid improve the induction of microspore embryogenesis and frequency of direct plant regeneration in pakchoi (*Brassica rapa* ssp. *chinensis* L.) [12]. Similarly, microspore-derived embryogenesis and regeneration of green wheat were activated by TSA treatment [14], suggesting that TSA induces an increase in histone acetylation and global alteration of gene expression. In addition, TSA accelerate the formation of embryogenic cell clusters from male gametophytes of *Arabidopsis thaliana*, which is recalcitrant to haploid embryo development in culture, likely through its inhibition of HDAC17 [13]. In *Arabidopsis*, TSA treatment also promotes embryogenic transition of explants as well as the development of root hair cells via the auxin-related pathway [15, 16].

Azacytidine (Aza) is a cytosine analog and DNA methyltransferase inhibitor commonly used in epigenetics studies [11]. The biological significance of DNA demethylation caused by Aza in plant regeneration has been examined in various plant species. Low concentrations of Aza promote callus proliferation and plant regeneration in anther wheat cultures [17]. In rapeseed and barley, the application of Aza induces microspore reprogramming, acquisition of totipotency, and initiation of embryogenesis, suggesting a potential role for DNA methylation in the repression of microspore reprogramming [18].

Based on the above-mentioned reports on chemicals that affect epigenetic regulation, we tested whether the three chemicals could be applied to improve efficiency of plant regeneration from protoplast cultures of other crop species and ultimately accelerate the development of genome-edited plants by application of the Cas9 protein-gRNA ribonucleoproteins (RNPs) transfection system. Therefore, in this study, the potential involvement of these chemicals in the initial processes of plant regeneration from the mitotic division of protoplast-derived cells in shoot regeneration was investigated in lettuce. We examined the efficiency of cell division, callus formation and adventitious shoot formation, and the morphology of regenerated plantlets from mesophyll protoplast cultures of tobacco. Western blotting and gene expression analysis using qRT-PCR were performed to test effects of TSA on histone acetylation and gene expression in tobacco.

## Materials and methods

### Plant materials and protoplast isolation

In this study, we used tobacco (*Nicotiana benthamiana*) and lettuce (*Lactuca sativa* L. ‘Cheongchima’). The seeds were sterilized with 70% ethanol for 3 min, 1% hypochlorite solution for 15 min; then, washed five times with distilled water. Sterilized seeds were inoculated on 1/2 Murashige and Skoog [19] solid medium containing 0.4 mg/L thiamine-HCl, 100 mg/L myo-inositol, 30 g/L sucrose, and 8 g/L Gelrite, pH 5.7. The tobacco seedlings were grown for 4 weeks at 25°C in a growth chamber under a 16 h light/8 h dark photoperiod (100–130 μmol/m^2^ s). The lettuce seedlings were grown for 1 week at 20°C in a growth chamber under a 16 h light/8 h dark photoperiod (100–130 μmol/m^2^ s). Protoplasts were isolated from tobacco and lettuce seedlings, as described previously, with some modifications [20, 21]. For protoplast isolation, the 4-week-old leaves of tobacco and 7 day-old cotyledons of seedlings were digested with 10 mL of enzyme solution (1% Viscozyme [Viscozyme L, Novozyme], 0.5% Celluclast [C2730, Novozyme], 0.5% Pectinex [33095, Novozyme], 9% mannitol, 3 mM MES [pH 5.7], cell protoplasts washing solution [22]) with shaking at 40 rpm for 4–6 h at 25°C in the dark. The protoplast mixture was then filtered through a 40 μm nylon cell strainer (352340, Falcon) and collected by centrifugation at 800 rpm for 5 min in a 15 mL round tube (41014, SPL). The supernatants were carefully removed using a sterile Pasteur pipette, and the remaining protoplasts were resuspended in W5 solution (2 mM MES [pH 5.7], 154 mM NaCl, 125 mM CaCl_2_, and 5 mM KCl) [23] and further centrifuged at 114× g for 5 min. The protoplast washing step was repeated more than twice. Finally, the protoplasts were resuspended in W5 solution and counted under a microscope using a hemocytometer. Tobacco mesophyll protoplasts were adjusted to a final density of 1 × 10^5^ protoplasts/mL of protoplast culture medium (B5 medium containing 60 g/L myo-inositol, 20 g/L sucrose, 0.5 mg/L 1-Naphthaleneacetic acid [NAA], and 2 mg/L 6-benzylaminopurine [BAP], pH 5.7). Lettuce mesophyll protoplasts were adjusted to a final density of 1 × 10^6^ protoplasts/mL of protoplast culture medium (MS medium containing 0.4 mg/L thiamine HCl, 100 mg/L myo-inositol, 30 g/L sucrose, 0.2 mg/L 2,4-dichlorophenoxyacetic acid [2,4-D], and 0.3 mg/L BAP, pH 5.7).

### TSA, NaB, and Aza treatment

After adjusting the protoplast density, mesophyll protoplasts were suspended in the protoplast culture medium and 2 mL of the suspension was distributed in 60 × 15 mm Petri dish (3002, Falcon). Each chemical was added to the 2 mL protoplast culture medium per dish immediately after the isolation of protoplasts and the culture dishes were incubated at 25°C in the dark. To prepare stock solutions of epigenetic chemicals, TSA (T8552, Sigma-Aldrich) and Aza (A2385, Sigma-Aldrich) were dissolved in dimethyl sulfoxide. NaB (303410, Sigma-Aldrich) was dissolved in distilled water. The stock solutions were sterilized through filtration and stored at -20°C until use. Treatment with epigenetic chemicals was performed in 10-fold increments; TSA was used in the concentration range of 0.1–10 μM for tobacco and 0.01–1 μM for lettuce. Aza was used at concentrations ranging from 0.01 to 1 μM, and NaB was used from 0.1 to 10 μM.

### Measurements of cell division efficiency from protoplast cultures

After TSA treatment, tobacco and lettuce protoplasts were incubated at 25°C in the dark for 4 weeks and subsequent cell division was periodically observed using an inverted microscope (Zeiss Primovert optical microscope). Cell division efficiency was measured by counting the number of protoplasts forming the cell plate during protoplast culture. Three independent experiments were performed on different days, and 100 cells were counted in one experiment by taking pictures of many cells sequentially for each treatment. The average number of cells per sample (100 cells) was determined from three biological replicates (300 cells in total).

### Formation of micro-callus, callus proliferation, and adventitious shoot formation from protoplast cultures

After 4 weeks of culture, the initial protoplast culture medium containing TSA, NaB or Aza was replaced with protoplast culture medium without TSA, NaB, or Aza. Tobacco cell colonies and micro-callus were observed under a microscope. To test the effects of TSA, NaB, and Aza on the formation of micro-callus from tobacco and lettuce mesophyll protoplasts, ImageJ software was used to quantify the micro-callus area and images of the micro-callus at 14 days after the treatments were performed. Images of micro-callus were also acquired using a Zeiss Primovert optical microscope. To quantify the micro-callus area, images of each experimental group from three biological replicates were analyzed. The protoplasts treated with each chemical were cultured at 25°C in the dark for 4 weeks; thereafter, 10 mL of the protoplast culture medium was added and the cultures were transferred to a 16 h light/8 h dark photoperiod (30 μmol/m^2^ s) and further cultured at 25°C with shaking at 50 rpm. After 4 weeks of culture, the micro-callus were transferred to the culture medium (MS medium containing 30 g/L sucrose, 6 g/L Gelrite, 0.1 mg/L NAA, 0.5 mg/L BAP, pH 5.7) for callus proliferation. For callus proliferation, three independent replicate experiments were performed, with four Petri dishes in each replicate. After about 4 weeks of culture on the callus proliferation medium, the number of cell aggregates with a diameter greater than 0.5 mm was counted using an automatic callus/colony counter (Quantica 500, Bioavlee).

To evaluate the effect of TSA on adventitious shoot formation in tobacco, protoplast-derived calluses were transferred to solid shoot induction medium. The shoot induction medium consisted of MS (Murashige and Skoog, 1962) with 0.4 mg/L thiamine HCl, 100 mg/L myo-inositol, 30 g/L sucrose, 2 mg/L Indole-3-acetic acid, and 1 mg/L BAP (pH 5.7). After 4 and 5 weeks of culture, the frequency of shoot formation (percentage of explants that formed adventitious shoots) was calculated. All culture combinations were evaluated in three replicates, and at least ten explants per Petri dish were analyzed per replicate.

### Plant regeneration from *N*. *benthamiana* protoplast-derived callus

To compare the morphological variations due to TSA treatment, whole plants were regenerated from protoplast-derived adventitious shoots. Adventitious shoots were carefully cut from the calluses and transferred to MS basal medium. The cultures were maintained in light (approximately 30 μmol m^-2^s^-1^ from cool-white fluorescent lamps with a 16 h photoperiod). After 4 weeks of incubation in light, the shoots were elongated and rooted. Regenerated rooting plantlets were transplanted into potting soil (vermiculite:perlite, 3:1 mixture) and maintained in a plastic container for 2 weeks. After emergence of new leaves from transplanted plants, acclimatized plants were transferred and maintained in a growth chamber (80 μmol m^-2^s^-1^ from cool-white fluorescent lamps with a 16 h photoperiod and 50–70% RH).

### Histone extraction and western blotting

Tobacco mesophyll protoplasts were cultured in a protoplast culture medium supplemented with 0, 0.1, 1, and 10 μM TSA. After 6 h of incubation with TSA, the protoplasts were transferred to 15 mL round tubes (41014, SPL) and harvested via centrifugation at 800 rpm for 5 min. Histone extraction was performed according to the manufacturer’s protocol (https://www.abcam.com/protocols/histone-extraction-protocol-for-western-blot), with some modifications. Histones were extracted with 0.2 N HCl overnight at 4°C. Supernatant was collected and neutralized with 0.2 volume of 1N NaOH. Histones were separated using 15% Sodium Dodecyl Sulfate Polyacrylamide Gel Electrophoresis (SDS-PAGE) and detected using a specific antibody against acetylated histone H3 (06–599, Merck Millipore) and histone H3 antibody (ab1791, Abcam) by Chemiluminescence system Fusion Solo S (Bilber, France). The ratio of histone H3 and acetylated histone H3 was calculated using the blot in software.

### RNAs isolation and RT-qPCR analysis

Total RNAs were extracted from each TSA-treated mesophyll protoplasts of tobacco using an aqueous kit (Invitrogen, AM9738) with a TURBO DNA-free kit (Applied Biosystems, AM1907). cDNA was synthesized using a high-capacity cDNA reverse transcription kit (4368814, Applied Biosystems). To investigate the transcript levels of *CDK*, *CYCD3-1*, and *WUS* in mesophyll protoplasts of tobacco, each primer was designed using the real-time PCR tool OligoAnalyzer 3.1 (Integrated DNA Technologies, Coralville, IA, USA). *CDK* and *NbWUS* gene information was obtained from Mlotshwa et al. (2006) [24]. qRT-PCR was performed using a SYBR Green Kit (DQ383-40h, BioFACT) to detect the transcript levels of each gene on a CFX96^TM^ Real-Time PCR Detection System (Bio-Rad). The primers used for qRT-PCR are as follows: 5′-CCAAAGGCCAATCGAGAAAAG-3′ and 5′-CAGAGTCCAGCACAATACCAG-3′ for *NbActin*; 5′-CAACTTGTTGCTGTAGCTTGTC-3′ and 5′-CAAACACATATCTTGGATCCTCAAC-3′ for *NbCYCD3-1*; 5′-TCACAAAACAAGCCCTCTTC-3′ and 5′-GGGTGAGCAAGCTAGTTAAC-3′ for *NbCDK*; 5′-CATCCGCTTCTTCTCATGGT-3′ and 5′-GAGTTGCCACCTGGTGATATT-3′ for *NbWUS*. *NbActin* was used as an internal control.

### Statistical analysis

The experiments were conducted using a completely randomized design with three replicates. To determine the significant difference in mean ranges, one-way ANOVA was performed using Origin software (Ver. 8; OriginLab Co, USA). The figures show the mean from biological replicates with standard deviation (SD) or standard error (SE).

## Results

### Effects of TSA, NaB, and Aza on cell division in lettuce protoplast cultures

**Fig 1:**
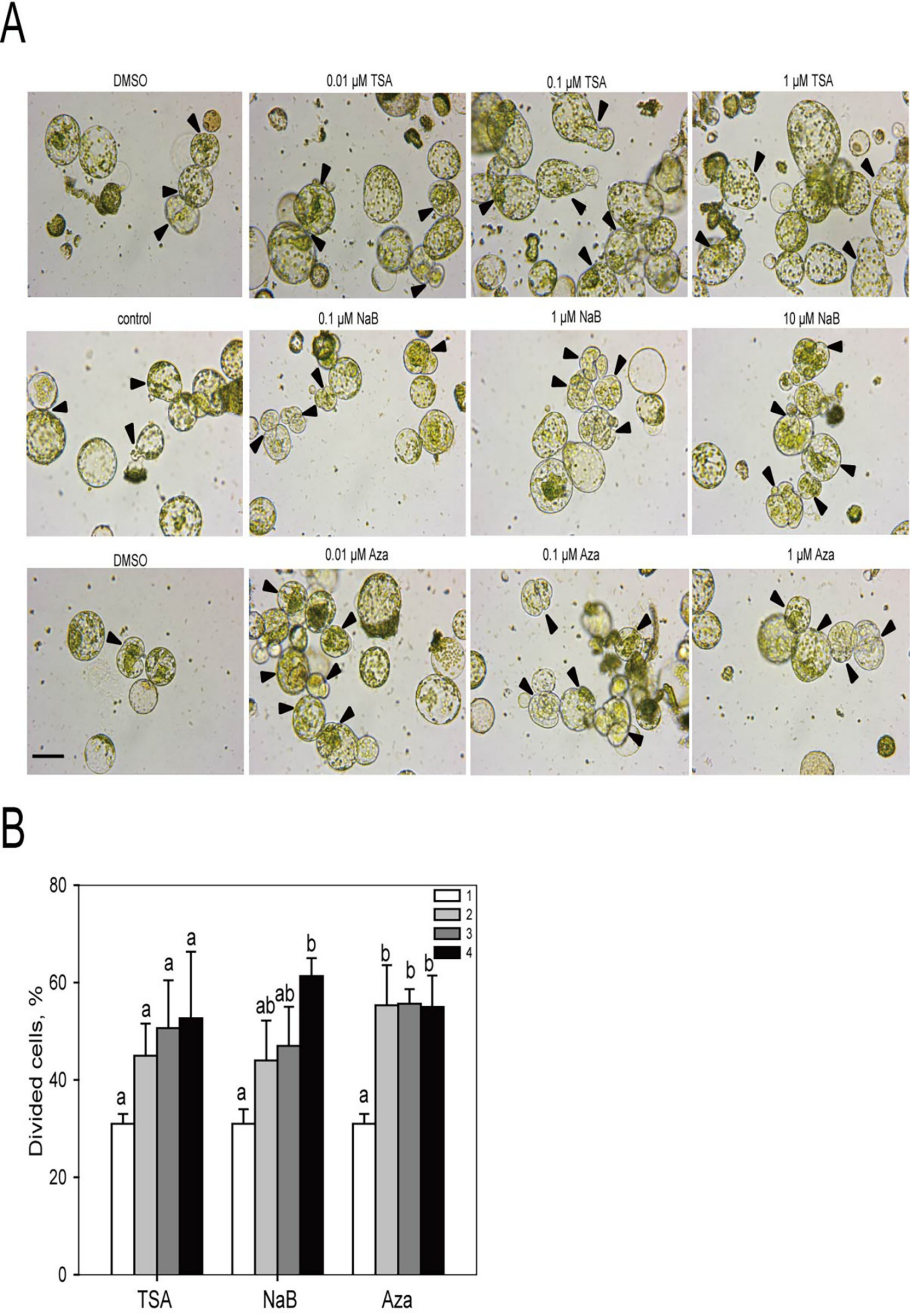
TSA, NaB, and Aza treatments increase the efficiency of cell division from lettuce mesophyll protoplasts. **A** Representative cell images. Arrowheads indicate dividing cells with a cell plate. Protoplasts were grown for 4 days after the treatments. One to four means ten-fold serial concentrations of the chemicals; the concentrations of TSA and Aza were 0, 0.01, 0.1, and 1.0 μM, and those of NaB were 0, 0.1, 1, and 10 μM. Scale bars: 50 μm. **B** The frequency of first cell division from mesophyll protoplasts was determined by counting the total numbers of cells with division. Protoplasts were grown for 4 days after the treatments. Bars represent means ± SE (n = 3) of three independent experiments using 300 cells. One to four means ten-fold serial concentrations of the compounds; the concentrations of TSA and Aza were 0, 0.01, 0.1, and 1.0 μM, and those of NaB were 0, 0.1, 1, and 10 μM. Different letters in the table indicate significant differences among the samples at a threshold of P < 0.05 [one-way ANOVA with Duncan’s test].

To examine the effects of TSA and NaB (inhibitors of HDACs) and Aza (an inhibitor of DNA methyltransferases) on initial cell division and differentiation, mesophyll protoplasts of lettuce were treated with several concentrations of TSA, NaB, and Aza. The concentration ranges of each chemical used in this experiment were based on previous reports on several other crops [12, 14, 17]. Interestingly, all three chemicals had a significant stimulatory effect on the initial cell division in the mesophyll protoplasts of lettuce (Fig 1). We observed actively dividing cells in newly formed cell plates after 4 days of incubation (indicated by arrows in Fig 1A). After 4 days of incubation, the efficiency of cell division from the mesophyll protoplasts of lettuce was examined (Fig 1B). Dividing cells were judged based on the presence of a cell plate (indicated by arrows in Fig 1), and the ratio of dividing protoplasts to the total number of protoplasts was examined. The cell division efficiency in control group was 31% after 4 days of incubation. Noteworthily, all three chemicals increased the cell division efficiency compared to the control group (Fig 1B). The cell division efficiency from 0.01, 0.1, and 1 μM of TSA treatments was 14%, 19.3%, and 20.3% higher than that of control group. In the TSA treatments, the cell division efficiency increased as the concentration of TSA increased. In case of NaB treatment, the cell division efficiency from 0.01, 0.1, and 1 μM NaB treatments was 13%, 16%, and 29.7%, respectively, higher than that of control group. Similar to TSA treatment, cell division efficiency after NaB treatment increased as the concentration of NaB increased. Aza also promoted cell division efficiency compared to the control. The cell division efficiency of all Aza treatments was approximately 24% higher than that of control group, regardless of Aza concentrations (0.01–1 μM). Based on the cell division efficiency, the optimal concentrations of TSA, Aza, and NaB were determined to be 1, 0.1, and 10 μM, respectively. However, the effects of each chemical on the increase in cell division from lettuce protoplasts varied. Interestingly, the stimulatory effect on cell division in TSA and NaB cells was concentration dependent. In contrast, the stimulatory effect of Aza on cell division did not show dose dependent manner at all.

**Fig 2:**
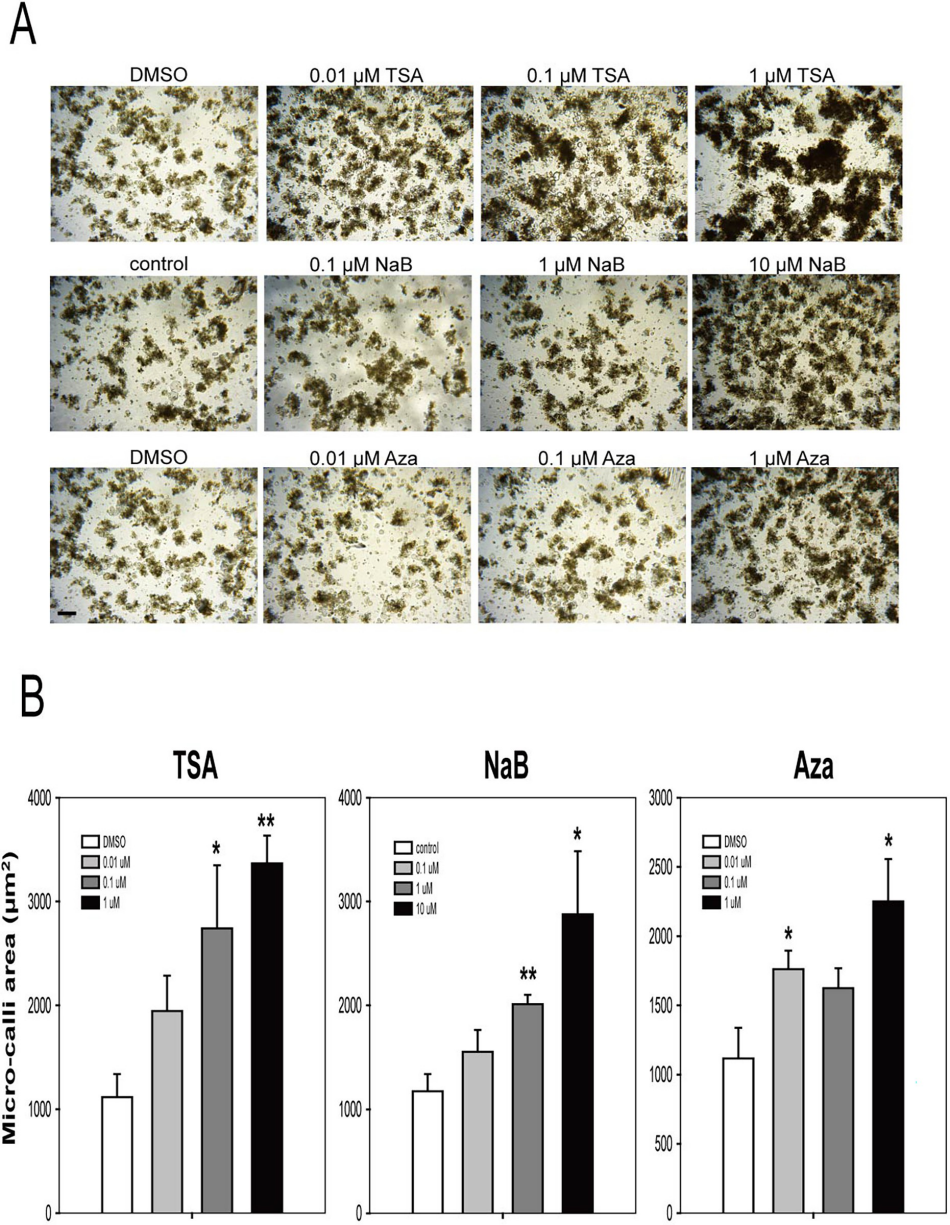
TSA, NaB, and Aza treatments accelerate micro-callus formation from protoplast cultures of lettuce. **A** Representative images of micro-callus grown for 14 days after the treatments. Scale bars: 100 μm **B** Quantitative measurements of the micro-callus area at 14 days after the treatments. The concentrations of TSA and Aza were 0, 0.01, 0.1, and 1.0 μM, and those of NaB were 0, 0.1, 1, and 10 μM. Bars represent means ± SE of three independent experiments and statistical significance was determined using a student’s *t*-test. **P≤0.01, *P≤0.05.

We further analyzed the effects of the three chemicals on micro-callus formation from protoplast cultures, which is the next step in plant regeneration. The actively dividing cell aggregates appeared dark brown under the microscope (Fig 2A). After 2 weeks of incubation, all three chemicals promoted the formation of micro-callus from mesophyll protoplasts of lettuce compared to the control (Fig 2B). TSA treatment promoted the formation of micro-callus at all concentrations (0.01–1 μM) in a concentration-dependent manner. NaB treatment also showed patterns similar to those of TSA. However, the increase in proliferation after NaB treatment was slightly lower than that after TSA treatment. In the case of the Aza treatment, many micro-calluses were formed at high concentrations. However, unlike TSA and NaB, Aza treatment did not show a concentration-dependent pattern. The promoting effect of TSA on micro-callus formation was higher than that of other chemicals. These results clearly show that the chemicals that characterize epigenetic modifications have stimulatory effects on initial cell division and micro-callus formation in lettuce.

### Effects of TSA, NaB, and Aza on the proliferation of protoplast-derived callus in lettuce

**Fig 3:**
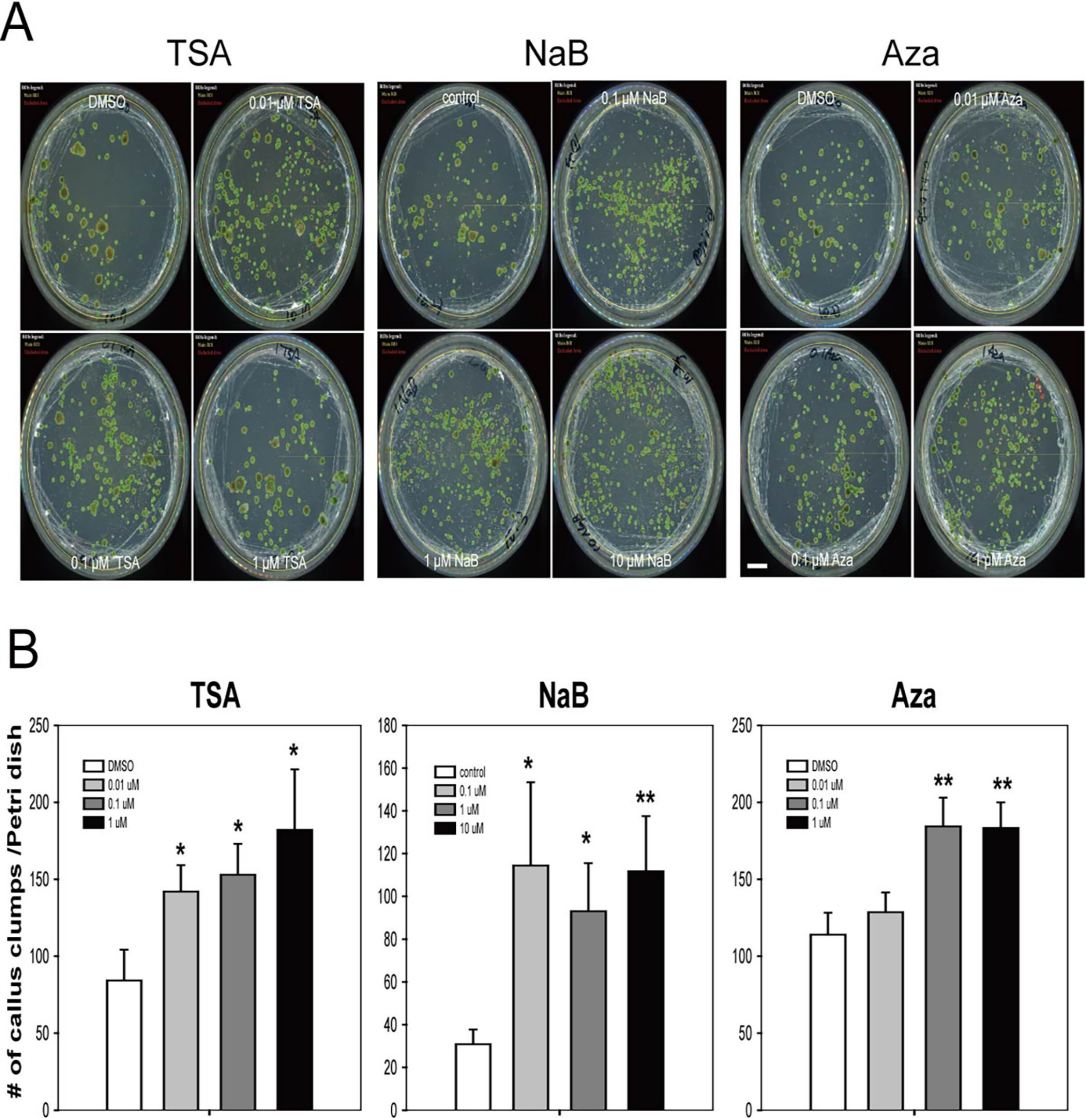
TSA, NaB, and Aza treatments enhance the callus proliferation efficiency. **A** Representative images of callus development after TSA treatment, NaB treatment, and Aza treatment during lettuce protoplast cultures. Scale bars: 1 cm **B** The number of callus clumps per petri dish after each treatment. The number of calluses, bigger in size than 0.5 mm in diameter, was counted automatically as shown in **A**. Bars represent means ± SE (n = 3) of three independent experiments. The concentrations of TSA and Aza were 0, 0.01, 0.1, and 1.0 μM, and those of NaB were 0, 0.1, 1, and 10 μM. Statistical significance was determined using a student’s *t-*test. **P≤0.01, *P≤0.05.

Next, we examined the effects of the three chemicals on callus proliferation (Fig 3). All three chemicals had a significant stimulatory effect on callus proliferation in the mesophyll protoplasts of lettuce. Most calluses (> 0.5 mm) turned green regardless of chemical type and concentrations (Fig 3A). The number of calluses per Petri dish increased by 1.69-, 1.82-, and 2.16-fold upon treatment with 0.01, 0.1, and 1 μM TSA, respectively (Fig 3B). Of note, the stimulatory effect of NaB on callus proliferation was much greater than that of TSA. The number of calluses per Petri dish from 0.1 to 10 μM NaB treatment was 3.17-, 3.02-, and 3.63-fold greater than that of the control group. These results indicate that NaB is the most effective in promoting callus proliferation. The stimulatory effect of Aza treatment on callus proliferation was lower than that of TSA and NaB. The number of calluses per Petri dish from 0.01, 0.1, and 1 μM of Aza treatment increased by 1.13-, 1.62-, and 1.61-fold, respectively, compared to that of the control group. The optimal concentrations of TSA, NaB, and Aza for callus proliferation were 1, 0.1, and 0.1 μM, respectively. These results clearly show that all three chemicals (TSA, NaB, and Aza) had a significant stimulatory effect on initial cell division and callus proliferation in mesophyll protoplast of lettuce, although the suitable concentration varied depending on the chemicals.

### Effects of TSA on cell division in tobacco protoplast cultures

**Fig 4:**
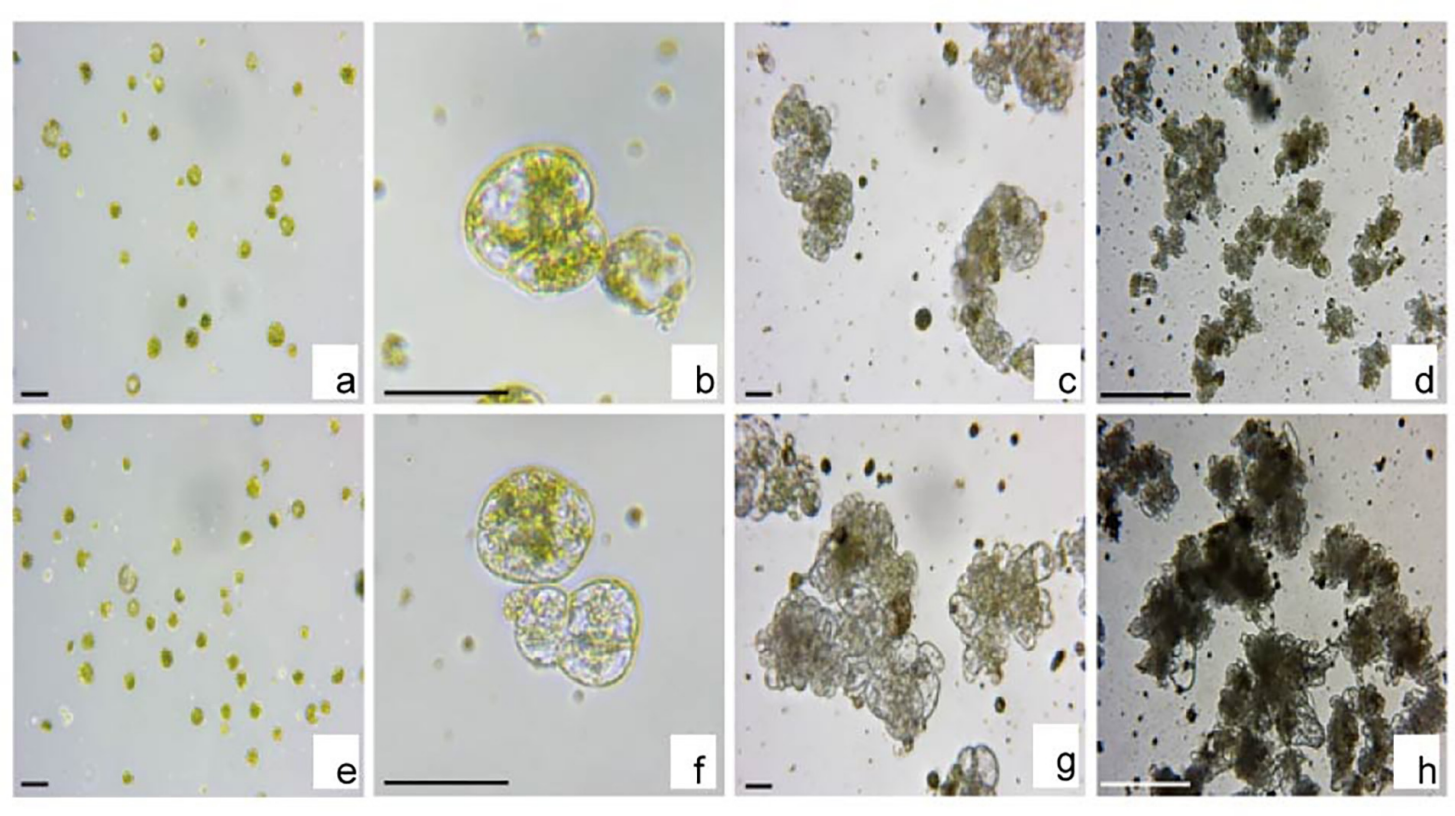
TSA increases the efficiency of cell division from mesophyll protoplasts of tobacco. **a** Freshly isolated mesophyll protoplasts of tobacco. **b** First cell division of protoplasts after 5 days of culture. **c** Cell colony formation after 2 weeks of culture. **d** Micro-callus formation after 4 weeks of culture. **e** Tobacco mesophyll protoplasts cultured on protoplast culture medium containing 1 μM TSA. **f** First and second cell division of the TSA-treated protoplasts after 5 days of culture. **g** Cell colony formation from TSA-treated protoplasts after 2 weeks of culture. **h** Micro-callus formation from TSA-treated protoplasts after 4 weeks of culture. Scale bars represent 50 μm (a-c, e-g) and 200 μm (d, h).

Of the three chemicals, TSA had the best concentration-to-concentration effect on micro-callus formation and callus proliferation efficiency, and we further investigated the effects of TSA on initial cell division and differentiation in other plant species. In our previous report, we found that TSA increased the efficiency of CRISPR/Cas9 gene editing owing to chromatin accessibility using lettuce and tobacco protoplasts [25]. In addition, TSA treatment substantially increased the level of histone H3 and H4 acetylation and the expression of cell division-related genes (*LsCYCD1-1*, *LsCYCD3-2*, *LsCYCD6-1*, and *LsCYCU4-1*) in lettuce protoplasts [25]. Therefore, we examined the effect of TSA on cell division in the mesophyll protoplasts of tobacco (Fig 4). Large quantities of mesophyll protoplasts were obtained from leaf explants after enzymatic digestion (Fig 4A), and the diameter of the freshly isolated protoplasts varied from 20 to 50 ㎛ (Fig 4A). The protoplasts underwent first cell division after 5 days of culture (Fig 4B) and many cell colonies formed through subsequent cell divisions after 2 weeks of culture (Fig 4C). Cell colonies reached a diameter of 200–400 ㎛ after 4 weeks of culture (Fig 4D). Mesophyll protoplasts of tobacco plants were treated with different concentrations of TSA (Fig 4E). The first cell division was observed after 3–5 days of culture (Fig 4F). The first and second cell division of the protoplasts were observed in the TSA-treated groups (Fig 4F). These results clearly show that TSA treatment accelerated cell division in the mesophyll protoplasts of tobacco. After 2–4 weeks of culture, many cell colonies formed after TSA treatment through subsequent cell divisions (Fig 4G and 4H). The size and number of cell colonies treated with TSA were much greater than those of cell colonies without TSA treatment (Fig 4H).

To examine the effect of TSA on cell divisions in tobacco mesophyll protoplasts, cell division efficiencies were investigated for each TSA treatment after 7 days of culture (Table 1). The cell division efficiency (first cell division + second cell division) of control treatment without TSA was 44.4%. The cell division efficiency in low concentration (0.1 μM) of TSA treatment was 47%, showing no remarkable difference from that of the control treatment. In particular, the highest cell division efficiency reached to 61.9% when tobacco mesophyll protoplasts were cultured on MS medium supplemented with 1 μM TSA. Moreover, the frequency of the second cell division from 1 μM TSA-treated mesophyll protoplasts was 2.2 times higher than that of protoplasts not treated with TSA (Table 1). These results clearly show that TSA promoted cell division of tobacco mesophyll protoplasts at optimal concentrations. However, the cell division efficiency decreased at high concentrations (10 μM) of TSA. This result indicates that a high concentration of TSA has an inhibitory effect on cell division. Taken together, these results provide evidence that TSA has a significant dose-dependent effect on cell division in tobacco protoplast culture.

**Table 1:** Effect of TSA on cell division and budding from tobacco mesophyll protoplasts after 7 days of culture.

Culture medium	Concentration of TSA (μM)	Frequency of first cell division^1^	Frequency of second cell division^2^
B56I2B05N	0	36.3 ± 5.5^ab^	8.1 ± 1.2^b^
	0.1	38.0 ± 7.4^bc^	9.0 ± 2.3^b^
	1	44.0 ± 9.6^c^	17.9 ± 1.9^c^
	10	29.5 ± 4.1^a^	5.8 ± 1.3^a^
B56I	0	0	0
	1	0	0

^1, 2^: The frequency of the first and second cell divisions from mesophyll protoplasts was determined by counting the total number of cells with division. Different letters in the table indicate significant differences among the samples at a threshold of P < 0.05 [one-way ANOVA with Duncan’s test].

To test whether TSA alone can promote cell division, tobacco mesophyll protoplasts were cultured in protoplast culture medium without plant hormones (S1 Fig and S1 Table). After 1 week of culture, mesophyll protoplasts could not be divided into the plant hormone-free medium (S1 Fig). Similar to the control treatment, 1 μM TSA was unable to promote cell division without plant hormones. However, the budding of protoplasts was increased in TSA-treated protoplasts (S1 Fig). These results indicate that the stimulatory effect of TSA on cell division requires the preferential aid of plant hormones.

### Effects of TSA on the proliferation of protoplast-derived callus and adventitious shoot formation in tobacco

**Fig 5:**
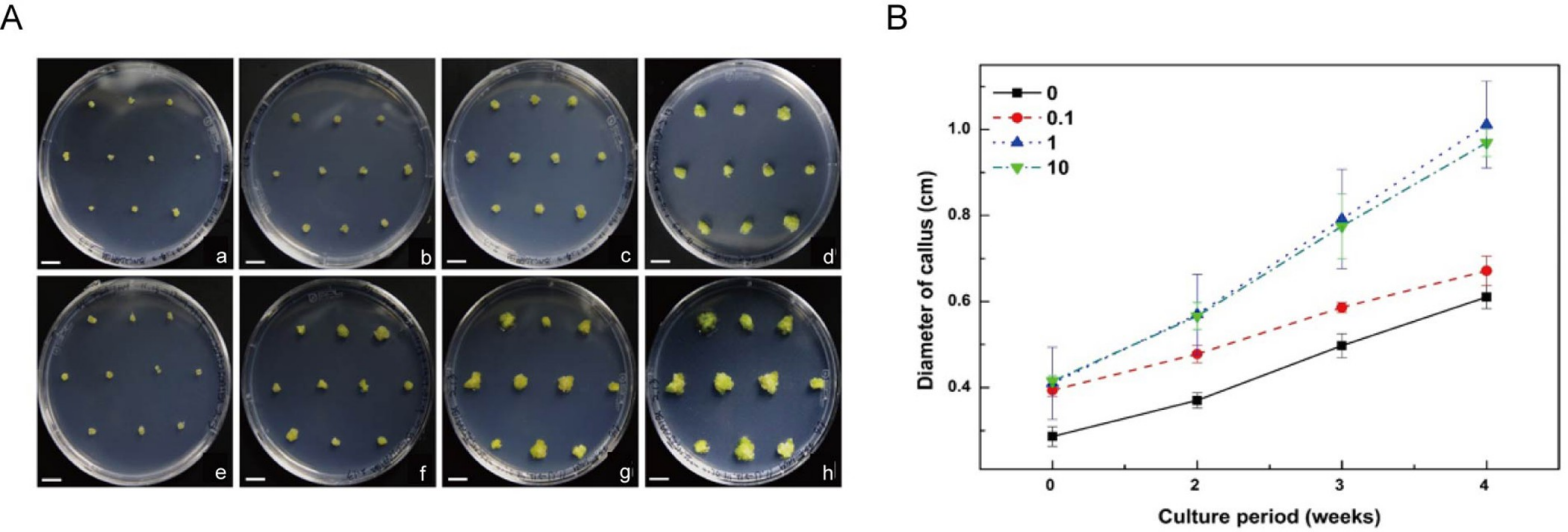
TSA treatments accelerate callus proliferation from mesophyll protoplasts of tobacco. **A** Representative images showing the difference in callus growth according to TSA treatment. Scale bars represent 1 cm. Callus formed from protoplasts without TSA treatment after one (**a**), two (**b**), three (**c**), and four (**d**) weeks of incubation. Callus formed from 1 μM TSA-treated protoplasts after one (**e**), two (**f**), three (**g**), and four (**h**) weeks of incubation. **B** Quantitative measurements of callus diameter. The concentrations of TSA treatments at protoplast culture stage were 0, 0.1, 1, and 10 μM. The diameter of callus was determined after incubation on solid callus proliferation medium without TSA treatment. Bars represent means ± SD of three independent experiments and statistical significance was determined using a student’s *t*-test. **P≤0.01, *P≤0.05.

To investigate whether TSA has a stimulatory effect on callus growth and adventitious shoot formation from callus, protoplast-derived cell colonies were transferred to callus inducing medium. First, the effect of TSA treatment on callus growth rate was examined (Fig 5). The diameter of callus from the control treatment was slightly smaller than that from the TSA treatment at the early stage of callus culture (Fig 5Aa and 5Ae). Initial diameter of callus was approximately 0.29 ± 0.02 cm in control treatment, whereas the one of callus treated with 0.1, 1, and 10 μM TSA was 0.39 ± 0.01, 0.41 ± 0.08 cm, and 0.41 ± 0.01 cm, respectively. The diameter of callus from control treatment was approximately 1.4 times smaller than that of TSA-treated callus in the initial step, regardless of TSA concentration (Fig 5B). After 2–4 weeks of culture, the diameter of callus reached approximately 0.61 ± 0.02 cm in control treatment (Fig 5B). The calluses treated with 1 and 10 μM TSA reached approximately 0.97–1.01 cm (Fig 5B). The diameter of callus treated with 1 and 10 μM TSA was approximately 1.7 times greater than that of control treatment after 4 weeks of culture (Fig 5B). These results clearly show that TSA had a dose-dependent stimulatory effect on callus proliferation in tobacco protoplast culture. However, low concentration (0.1 μM) of TSA treatment did not show substantial acceleration of callus growth (Fig 5B). Similar to the control callus (Fig 5Ab–5Ad), all TSA-treated calluses turned green regardless of TSA concentration (Fig 5Af–5Ah).

**Fig 6:**
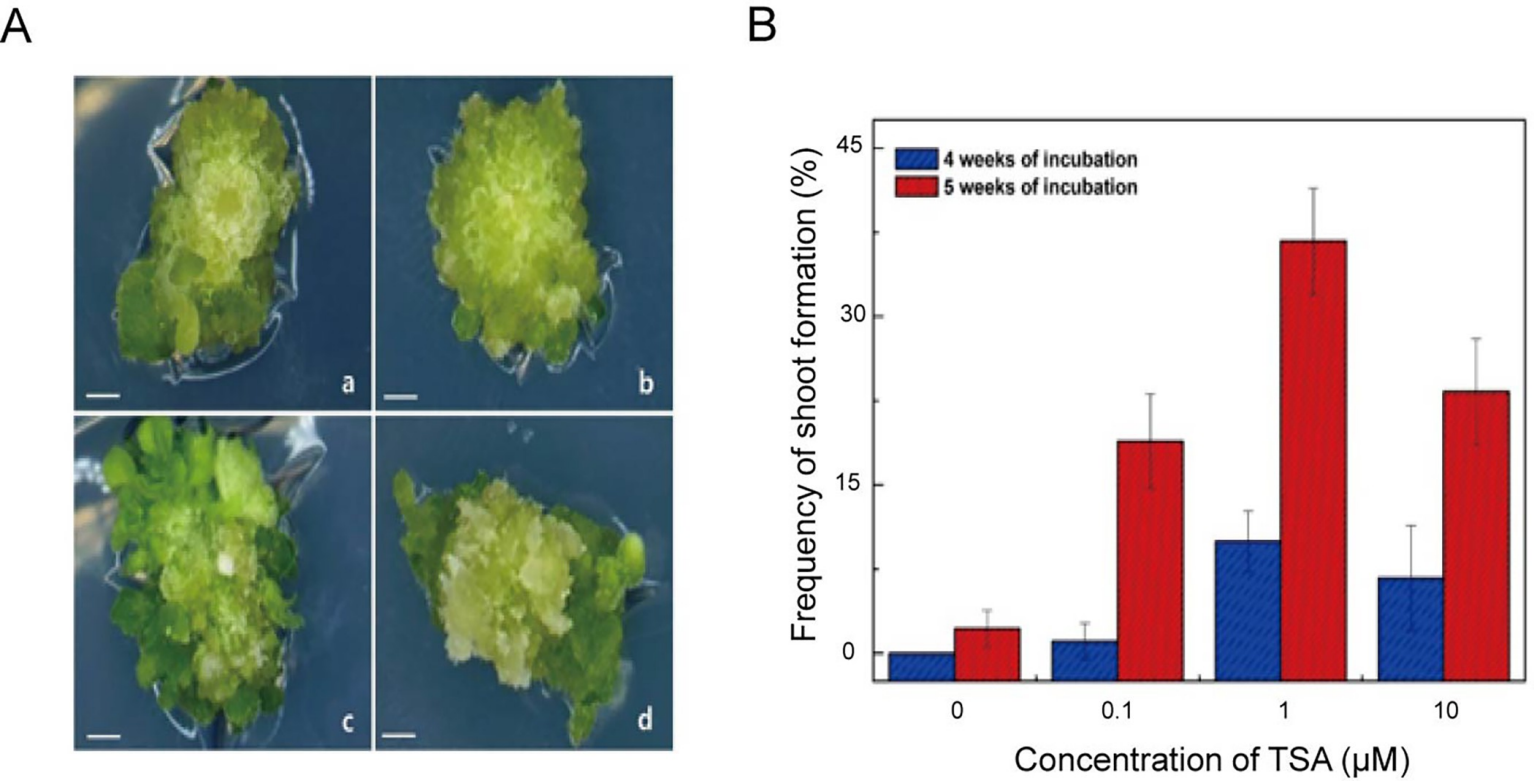
TSA treatments enhance the efficiency of adventitious shoot formation from protoplast-derived calluses. **A** Representative images showing the difference in shoot formation according to TSA treatment. Formation of numerous shoots was observed after 5 weeks of incubation on shoot induction medium. Callus formed from protoplasts without TSA treatment (**a**), Callus formed from 1 μM TSA-treated protoplasts (**b-d**). Scale bars represent 1 mm. **B** Quantitative measurements of frequency of shoot formation. The frequency of shoot formation from green calluses was determined after 4 and 5 weeks of incubation on shoot induction medium without TSA treatment. Bars represent means ± SD (n = 3) of three independent experiments. The concentrations of TSA were 0, 0.1, 1, and 10 μM. Statistical significance was determined using a student’s *t*-test. **P≤0.01, *P≤0.05.

Green calluses were transferred to shoot inducing medium for adventitious shoot formation. Numerous adventitious shoots were formed from TSA-treated green calluses after 4 weeks of incubation on shoot inducing medium (Fig 6). Adventitious shoots did not form on the green calluses in the control group during the same incubation period. In contrast to TSA treatments, only a shoot was formed in the control group after one more week of incubation (Fig 6Aa). Also, the frequency of adventitious shoot formation in control group was 2.2% after 5 weeks of incubation. However, the frequency of adventitious shoot formation in 0.1, 1, and 10 μM TSA treatments was 18.9%, 36.7%, and 23.3%, respectively (Fig 6B). The frequency of adventitious shoot formation by TSA treatment was 8.5 to 16.5 times higher than that in the control group. The number of adventitious shoots per callus ranged after 5 to 12 by TSA treatments after 5 weeks of culture (Fig 6Ab–6Ad). These results clearly show that the frequency of adventitious shoot formation was remarkably increased by TSA treatment in a dose-dependent manner. In addition, the incubation period for shoot formation from the TSA treatments was shortened by more than 1 week compared to the control group. However, the frequency of adventitious shoot formation at higher concentration (10 μM) of TSA treatment was slightly decreased.

**Fig 7:**
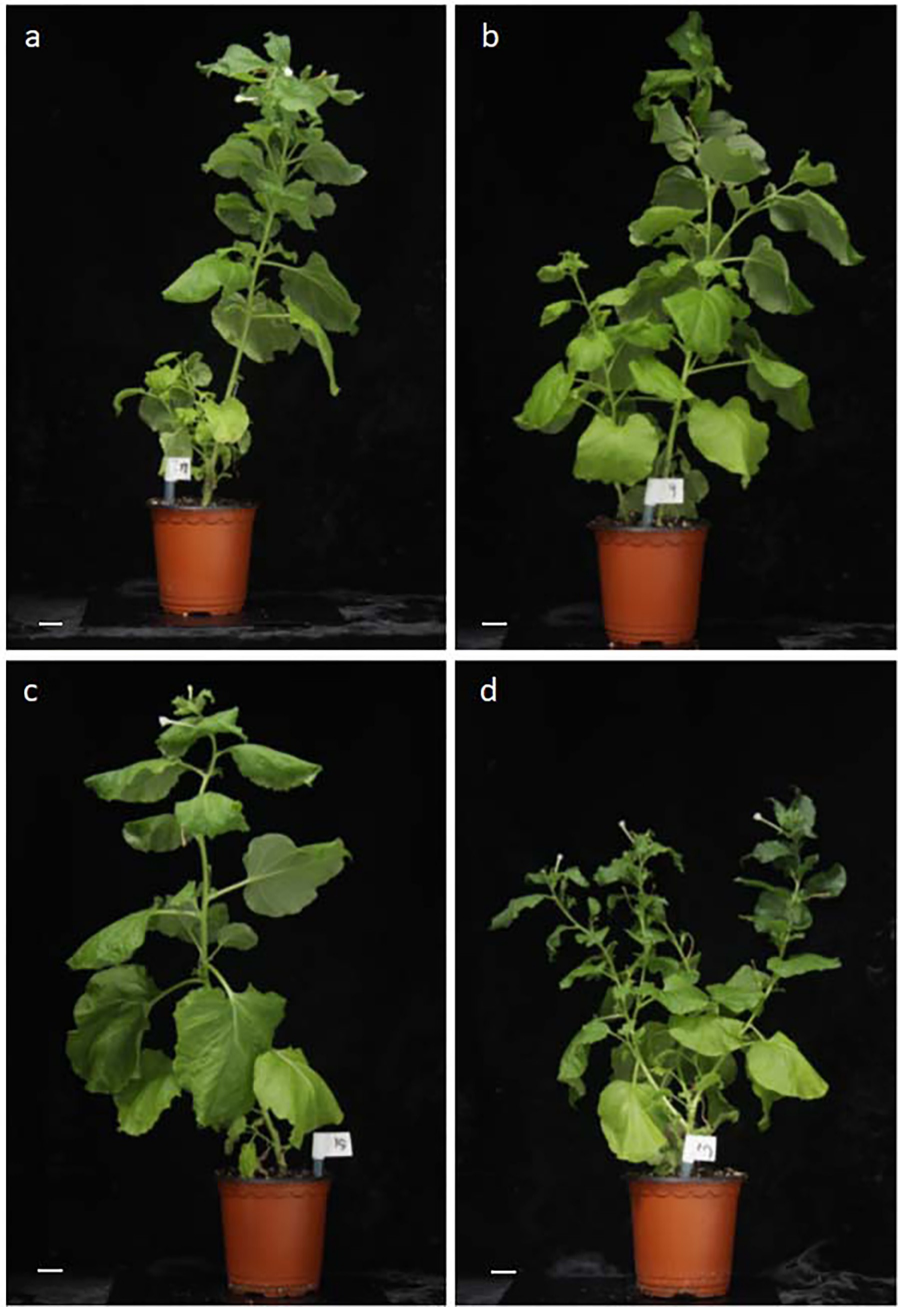
Regenerated tobacco plants from TSA-treated protoplasts did not show morphological changes compared to control. Morphology of the regenerated plantlets with rooting after TSA treatment in tobacco protoplast cultures. Representative images showing no difference in tobacco plants formed from protoplasts without TSA treatment (**a**, **b**) and from 1 μM TSA-treated protoplasts (**c, d**). Scale bars: 2 cm.

To compare the morphological variations due to TSA treatment, whole plants were regenerated from protoplast-derived adventitious shoots. Regenerated plantlets with rooting were transplanted into potting soil, and the morphology of the regenerated plantlets after TSA treatment in tobacco protoplast cultures were examined. There was no substantial difference in the appearance of the generated plantlets between tobacco plants formed from protoplasts without TSA treatment and from 1μM TSA-treated protoplasts (Fig 7). The regenerated tobacco plants from TSA-treated protoplasts did not show morphological changes compared with the control. These results suggest that epigenetic regulator (s) could be applied to increase the efficiency of plant regeneration from protoplasts without side effects.

### Effect of TSA on histone acetylation and gene expression in tobacco

**Fig 8:**
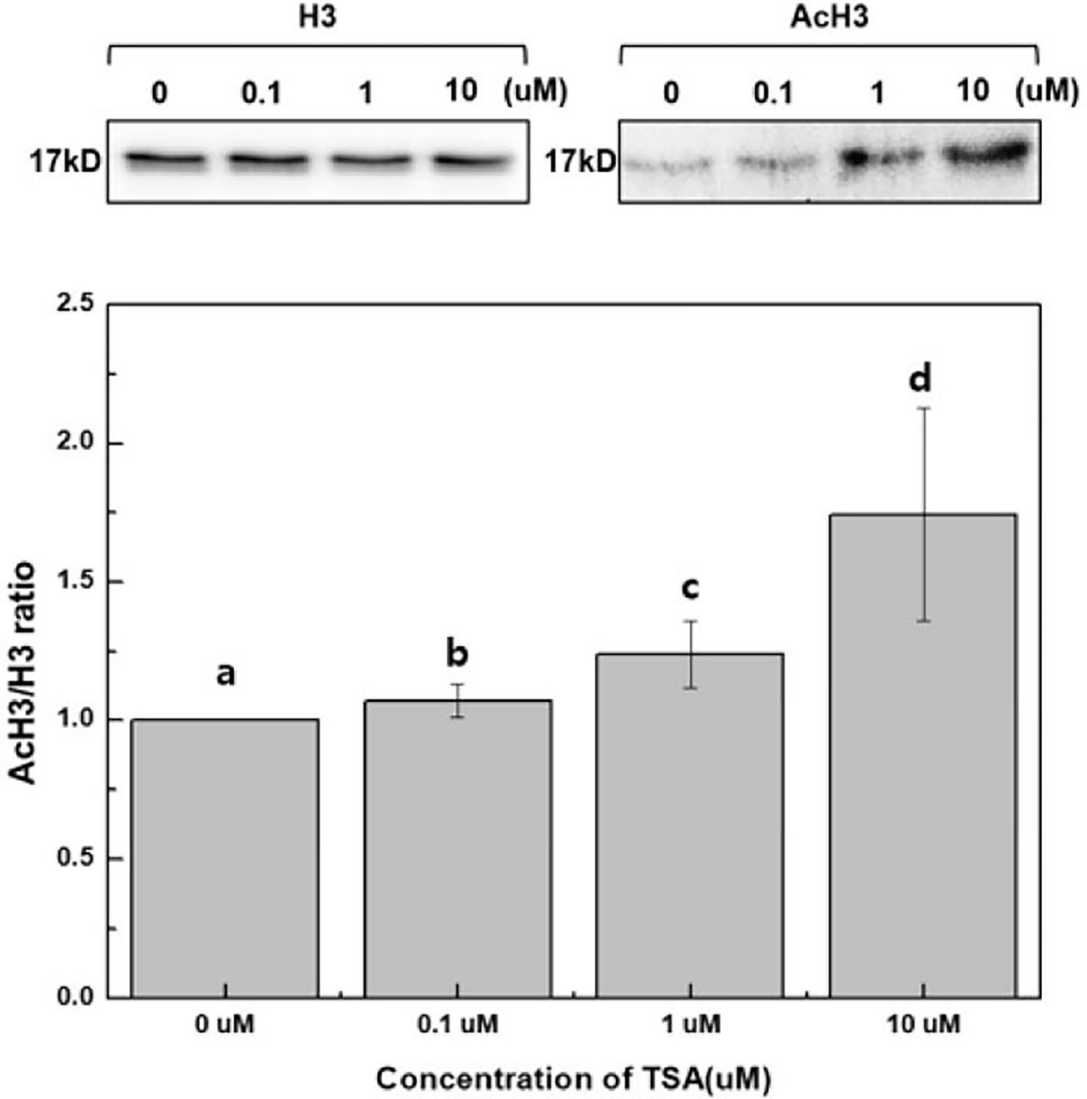
The effect of TSA on histone H3 acetylation level from tobacco protoplasts. Total protein extracts were obtained from tobacco protoplasts after 6 h of TSA treatments. The level of H3 histone acetylation was determined via western blot analysis using anti-H3 and anti-AcH3 antibodies. Different letters on the bars indicate significant differences between each treatment. H3 = histone H3 antibody; AcH3 = acetylated histone H3 antibody.

TSA has been reported to increase total histone H3 acetylation in plants [25, 26]. To investigate the effect of TSA on histone acetylation in protoplast cultures, western blot analysis was performed on *N*. *benthamiana* protoplasts after TSA treatment using antibodies directed against H3 and AcH3 (Fig 8). After 6 h of TSA treatment, histone H3 acetylation in *N*. *benthamiana* protoplasts was dramatically increased in a dose-dependent manner compared to the control group. These results indicate that TSA increases histone H3 acetylation levels from tobacco mesophyll protoplasts.

**Fig 9:**
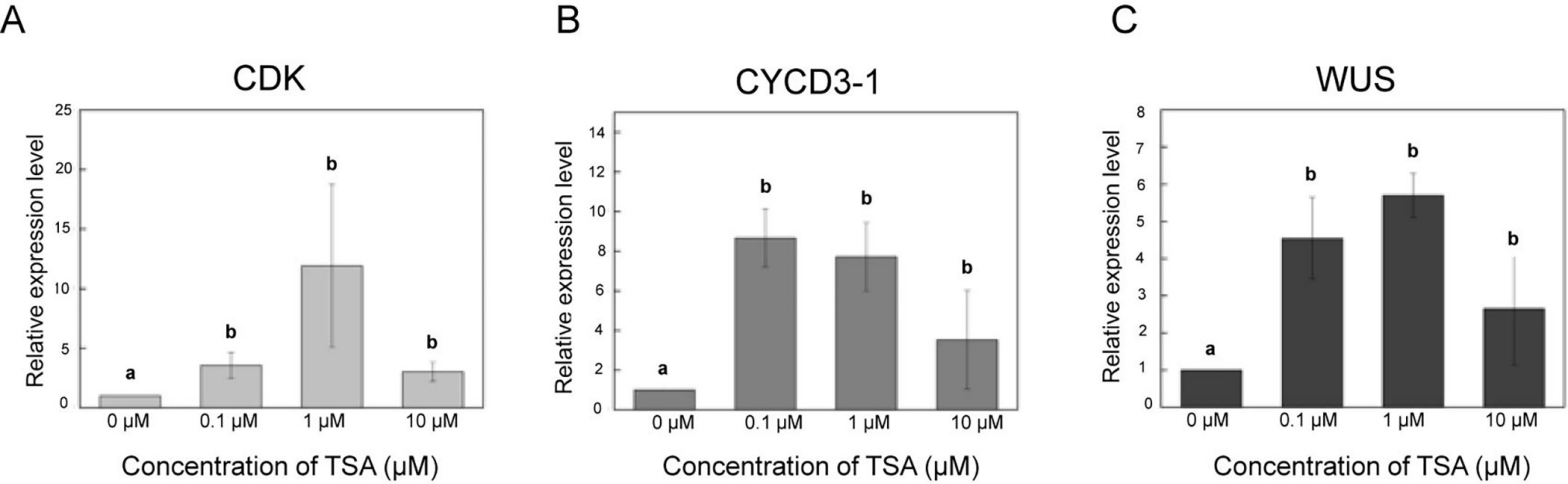
The effect of TSA on expression of *CDK*, *CYCD3-1*, and *WUS* genes from tobacco protoplasts. The relative gene expression levels of *CDK* (**A**), *CYCD3-1* (**B**), and *WUS* (**C**) in tobacco protoplasts. qRT-PCR was performed with total RNA extracted from tobacco protoplasts after TSA treatment for 6 h. Bars represent means ± SE (n = 3) of independent experiments. Different letters on the bars indicate significant differences between each treatment (ANOVA with Duncan’s test, p < 0.05).

We already observed an increase in cell division efficiency (Fig 4), callus proliferation (Fig 5), and adventitious shoot formation (Fig 6) in TSA-treated protoplasts compared to the control group during protoplast culture. Therefore, we investigated whether the increase in the stimulatory effect of TSA on cell division and adventitious shoot formation was caused by an increase of gene expression. We examined the expression of Cyclin-dependent kinase (*CDK*), Cyclin D3-1 (*CYCD3-1*), cell cycle regulatory genes, and WUSCHEL (*WUS*), a transcription factor that plays a central role for the maintenance of the stem cell niche in the shoot apical meristem [27–31]. TSA treatment substantially increased the expression of *CDK*, *CYCD3-1* and *WUS* (Fig 9). The gene expression of *CDK* and *WUS* was highest in the 1 μM TSA treatment, whereas gene expression of *CYCD3-1* was highest in the 0.1 μM TSA treatment (Fig 9). At a high concentration (10 μM) of TSA, the gene expression of *CDK*, *CYCD3-1* and *WUS* was slightly decreased. However, there was no significant difference in gene expression among the different TSA concentrations. TSA increased histone H3 acetylation levels in tobacco mesophyll protoplasts (Fig 8). TSA treatment substantially increased the expression of *CDK*, *CYCD3-1* and *WUS* (Fig 9). Combining these results, the increase in cell division efficiency, callus proliferation, and adventitious shoot formation after TSA treatment are directly related to changes in gene expression caused by the modification of histone acetylation levels.

## Discussion

Innovations in plant breeding are being developed using several technologies, including protoplast-based technologies such as electro-fusion, micronucleus transfer, direct DNA uptake, and CRISPR/Cas9-mediated genome editing [32, 33]. To achieve this goal, many studies have been conducted to optimize and maximize the plant regeneration efficiency of protoplast cultures. Here, we observed that the three chemicals (TSA, NaB, and Aza) promoted cell division and callus proliferation in lettuce mesophyll protoplasts. And we examined the effects of TSA on cell division, callus proliferation, adventitious shoot formation, and histone acetylation in tobacco mesophyll protoplasts. Establishing a more efficient and reproducible plant regeneration process from protoplast cultures will enable the application of powerful and commercial protoplast-based technologies. In particular, the RNP system can be applied to effectively regenerate new plants and would facilitate the development of non-genetically modified cultivars.

Epigenetic regulation is important for plant regeneration [5, 34, 35]. Epigenetic reprogramming during de novo organogenesis in plant tissue cultures is well-known [5]. It was only recently reported that TSA treatment increases micro-callus formation and callus development in *Arabidopsis* protoplasts [27]. However, the effects of chemicals on the regeneration of crop plants from tobacco and lettuce protoplast culture has not been studied. The low frequency of plant regeneration from protoplast culture is one of the main limitations of its routine application in crop development using protoplast technologies [32]. Our results indicate that adding HDAC or DNA methyltransferase inhibitors to protoplast culture medium can effectively improve cell division and callus proliferation during plant regeneration. In this study, we demonstrated that the addition of three chemicals (TSA, NaB, and Aza) into protoplast culture medium promoted the subsequent steps, that is, cell division, micro-callus formation, and callus formation. Therefore, the addition of epigenetic regulators could be potentially used as a new alternative to increase the efficiency of in vitro cellular differentiation in several economic crops.

Acetylation of lysine residues on histones results in the removal of their positive charge, which alters histone-histone and histone-DNA interactions and changes the accessibility of DNA to chromatin-binding proteins [36]. Hence, histone acetylation is closely associated with the open chromatin state and activation of gene transcription [37]. We have previously demonstrated that TSA has a stimulatory role in increasing the efficiency of genome editing and causes an increase in global histone H3 and H4 acetylation in lettuce protoplasts [25]. In this study, TSA increased histone H3 acetylation and expression of *CDK*, *CYCD3-1*, and *WUS* genes in tobacco protoplasts. Thus, we suggest that genes that should be blocked by histone deacetylation during the early stage of cell division is induced by TSA treatment, and these are likely to help in later callus formation and shoot regeneration. To the best of our knowledge, the key mechanism for promoting callus formation and plant regeneration is facilitation of cell division.

The stimulatory effects of two HDAC inhibitors (TSA and NaB) on cell division and callus proliferation from mesophyll protoplasts of lettuce and tobacco showed a linear correlation in a dose-dependent manner. However, the effects of the DNA methyltransferase inhibitor (Aza) were not dose-dependent. TSA inhibited root growth in *Populus trichocarpa* [38]. TSA decreased the length of regenerated roots and root number in a dose-dependent manner. Martinez et al (2021) recently reported that HDAC inhibitors play a role in the embryogenic response of grapevine explants in a dose-dependent manner [39]. The effect of TSA treatment on protoplast-derived cell division may be different from that on root and somatic embryo development. Therefore, it is important to choose the optimum concentration of HDAC inhibitors by considering each effect when re-differentiating plants, because the best effect for each concentration is different. As reported in previous studies [5, 35], because global and local changes in chromatin structure during callus proliferation and *de novo* organogenesis are dramatically different, the effects of these chemicals on each step of plant regeneration must be different. It would also be interesting to study the effects of these chemicals on the induction of other organs in a new plant.

Mutations in HDAC or DNA methyltransferase resulted in a phenotype similar to that observed in TSA-treated plants with respect to plant regeneration [10, 40]. The three inhibitors have been suggested to perhaps inhibit specific HDACs or DNA methyltransferases [11]. In *Arabidopsis*, an HDA6/HDA19 double-repression line similarly showed growth arrest and embryo-like structures on mature leaves without an HDAC inhibitor, suggesting that inhibition of HDA19, which functions redundantly with HDA6 in promoting normal post-germination growth, mediates TSA-induced growth arrest [40]. TSA also alters the development of root hair cells in *Arabidopsis*, likely through its inhibition of HDA18, because mutation of HDA18 resulted in a phenotype similar to that observed in TSA-treated wild-type plants [41]. Therefore, each chemical promotes plant regeneration through a distinct route, and at the same concentration, the effects of cell division, callus proliferation, and shoot regeneration appear to be different. The exact mechanisms by which the HDAC inhibitors TSA and NaB and the DNA methyltransferase inhibitor Aza mediate chromatin remodeling for plant regeneration from protoplasts remain to be elucidated.

In this study, we tested whether TSA alone could promote cell division in tobacco mesophyll protoplasts. When we cultured tobacco mesophyll protoplasts on protoplast culture medium without plant hormones after TSA treatment, mesophyll protoplasts could not divide after 1 week of culture (S1 Fig). Similar to the control treatment, 1 μM TSA did not promote cell division in the hormone-free medium. However, budding of protoplasts increased in TSA-included protoplast culture medium (S1 Fig). These results indicate that the stimulatory effect of TSA on cell division requires the preferential aid of plant hormones. The epigenetic regulator(s) seem to help in securing totipotency by reprogramming mesophyll protoplasts with external hormone treatment. Hormone-induced chromatin-dependent cell fate changes occur, and increased chromatin accessibility by treatment with epigenetic regulator(s) affects stochastic expression, giving rise to accelerated mesophyll protoplast regeneration. Although differentiated cells can be transformed into other cells (i.e., transdifferentiation) directly, it seems difficult to control by treating with a single epigenetic regulator because the entire epigenome must be simultaneously and complexly regulated.

To compare the morphological variations caused by TSA treatment, whole plants were regenerated from protoplast-derived adventitious shoots (Fig 7). Regenerated plantlets with rooting were transplanted into potting soil, and we examined the morphology of the regenerated plantlets after TSA treatment in tobacco protoplast cultures. We observed no significant differences in the appearance of the generated plantlets between tobacco plants formed from protoplasts without TSA treatment and from 1 μM TSA-treated protoplasts. These results suggest that we could apply epigenetic regulator(s) to increase the efficiency of plant regeneration from protoplasts without side effects. Recently, the development of genome-edited crops using the CRISPR/Cas9 system has attracted attention for reducing the risk associated with genetically modified organisms containing foreign DNA sequences [32, 42]. To achieve this goal, whole plant regeneration from a single protoplast is essential for the application of the CRISPR/Cas9 system. The increase in cell division and differentiation efficiency from protoplast cultures has helped us to rapidly develop useful genome-edited crops.

## Supporting information

S1 Raw images Raw western blot data using anti-H3 and anti-AcH3 antibodies in Fig 8. Total protein extracts were obtained from tobacco protoplasts after 6 h of TSA treatments. The level of H3 histone and H3 histone acetylation were determined via western blot analysis using anti-H3 and anti-AcH3 antibodies.
(PDF)

S1 Fig Effect of TSA on cell budding from mesophyll protoplasts of *N*. *benthamiana*. **A** Freshly isolated mesophyll protoplasts cultured on B56I medium without TSA. **B** After 7 days of culture on B56I medium without TSA. **C** Freshly isolated mesophyll protoplasts cultured on B56I medium with 1 uM TSA. **D** After 7 days of culture on B56I medium with 1 uM TSA. Cell budding was observed after 7 days of culture. Scale bars represent 50 μm.
(DOCX)

S1 Table Effect of TSA on cell budding from mesophyll protoplasts of *N*. *benthamiana* after 7 days of culture. (DOCX)

## References

[1] Fehér A. Callus, Dedifferentiation, Totipotency, Somatic Embryogenesis: What These Terms Mean in the Era of Molecular Plant Biology? 2019;10. doi: 10.3389/fpls.2019.00536 31134106 PMC6524723

[2] Su YH, Tang LP, Zhao XY, Zhang XS. Plant cell totipotency: Insights into cellular reprogramming. J Integr Plant Biol. 2021;63(1):228–43. Epub 2020/05/22. doi: 10.1111/jipb.12972 . 32437079

[3] Jeong YY, Lee HY, Kim SW, Noh YS, Seo PJ. Optimization of protoplast regeneration in the model plant Arabidopsis thaliana. Plant Methods. 2021;17(1):21. Epub 2021/02/25. doi: 10.1186/s13007-021-00720-x ; PubMed Central PMCID: PMC7901198. 33622383 PMC7901198

[4] Ikeuchi M, Shibata M, Rymen B, Iwase A, Bagman AM, Watt L, et al. A Gene Regulatory Network for Cellular Reprogramming in Plant Regeneration. Plant Cell Physiol. 2018;59(4):765–77. Epub 2018/02/21. doi: 10.1093/pcp/pcy013 ; PubMed Central PMCID: PMC6018650. 29462363 PMC6018650

[5] Lee K, Seo PJ. Dynamic Epigenetic Changes during Plant Regeneration. Trends Plant Sci. 2018;23(3):235–47. Epub 2018/01/18. doi: 10.1016/j.tplants.2017.11.009 . 29338924

[6] Karlic R, Chung HR, Lasserre J, Vlahovicek K, Vingron M. Histone modification levels are predictive for gene expression. Proc Natl Acad Sci U S A. 2010;107(7):2926–31. Epub 2010/02/06. doi: 10.1073/pnas.0909344107 ; PubMed Central PMCID: PMC2814872. 20133639 PMC2814872

[7] Us-Camas R, Rivera-Solís G, Duarte-Aké F, De-la-Peña C. In vitro culture: an epigenetic challenge for plants. Plant Cell, Tissue and Organ Culture (PCTOC). 2014;118(2):187–201. doi: 10.1007/s11240-014-0482-8

[8] Bannister AJ, Kouzarides T. Regulation of chromatin by histone modifications. Cell Res. 2011;21(3):381–95. Epub 2011/02/16. doi: 10.1038/cr.2011.22 ; PubMed Central PMCID: PMC3193420. 21321607 PMC3193420

[9] Tian L, Fong MP, Wang JJ, Wei NE, Jiang H, Doerge RW, et al. Reversible histone acetylation and deacetylation mediate genome-wide, promoter-dependent and locus-specific changes in gene expression during plant development. Genetics. 2005;169(1):337–45. Epub 2004/09/17. doi: 10.1534/genetics.104.033142 ; PubMed Central PMCID: PMC1448893. 15371352 PMC1448893

[10] Li W, Liu H, Cheng ZJ, Su YH, Han HN, Zhang Y, et al. DNA methylation and histone modifications regulate de novo shoot regeneration in Arabidopsis by modulating WUSCHEL expression and auxin signaling. PLoS Genet. 2011;7(8):e1002243. Epub 2011/08/31. doi: 10.1371/journal.pgen.1002243 ; PubMed Central PMCID: PMC3158056. 21876682 PMC3158056

[11] Zhang H, Wang B, Duan CG, Zhu JK. Chemical probes in plant epigenetics studies. Plant Signal Behav. 2013;8(9). Epub 2013/07/11. doi: 10.4161/psb.25364 ; PubMed Central PMCID: PMC4002629. 23838953 PMC4002629

[12] Zhang L, Zhang Y, Gao Y, Jiang X, Zhang M, Wu H, et al. Effects of histone deacetylase inhibitors on microspore embryogenesis and plant regeneration in Pakchoi (Brassica rapa ssp. chinensis L.). Scientia Horticulturae. 2016;209:61–6. doi: 10.1016/j.scienta.2016.05.001

[13] Li H, Soriano M, Cordewener J, Muino JM, Riksen T, Fukuoka H, et al. The histone deacetylase inhibitor trichostatin a promotes totipotency in the male gametophyte. Plant Cell. 2014;26(1):195–209. Epub 2014/01/28. doi: 10.1105/tpc.113.116491 ; PubMed Central PMCID: PMC3963568. 24464291 PMC3963568

[14] Jiang F, Ryabova D, Diedhiou J, Hucl P, Randhawa H, Marillia EF, et al. Trichostatin A increases embryo and green plant regeneration in wheat. Plant Cell Rep. 2017;36(11):1701–6. Epub 2017/07/29. doi: 10.1007/s00299-017-2183-3 . 28752355

[15] Wojcikowska B, Botor M, Moronczyk J, Wojcik AM, Nodzynski T, Karcz J, et al. Trichostatin A Triggers an Embryogenic Transition in Arabidopsis Explants via an Auxin-Related Pathway. Front Plant Sci. 2018;9:1353. Epub 2018/10/03. doi: 10.3389/fpls.2018.01353 ; PubMed Central PMCID: PMC6146766. 30271420 PMC6146766

[16] Xu CR, Liu C, Wang YL, Li LC, Chen WQ, Xu ZH, et al. Histone acetylation affects expression of cellular patterning genes in the Arabidopsis root epidermis. Proc Natl Acad Sci U S A. 2005;102(40):14469–74. Epub 2005/09/24. doi: 10.1073/pnas.0503143102 ; PubMed Central PMCID: PMC1242287. 16176989 PMC1242287

[17] Belchev I, Tchorbadjieva M, Pantchev IJBJPP. Effect of 5-azacytidine on callus induction and plant regeneration potential in anther culture of wheat (Triticum aestivum L.). 2004;30(1–2):45–50.

[18] Solís M-T, El-Tantawy A-A, Cano V, Risueño MC, Testillano PSJFips. 5-azacytidine promotes microspore embryogenesis initiation by decreasing global DNA methylation, but prevents subsequent embryo development in rapeseed and barley. 2015;6:472. 10.3389/fpls.2015.00472 PMC4479788 26161085

[19] Murashige T, Skoog F. A revised medium for rapid growth and bioassays with tobacco tissue cultures. Physiologia plantarum. 1962;15:473–97.

[20] Woo JW, Kim J, Kwon SI, Corvalan C, Cho SW, Kim H, et al. DNA-free genome editing in plants with preassembled CRISPR-Cas9 ribonucleoproteins. Nat Biotechnol. 2015;33(11):1162–4. Epub 2015/10/20. doi: 10.1038/nbt.3389 . 26479191

[21] Yoo SD, Cho YH, Sheen J. Arabidopsis mesophyll protoplasts: a versatile cell system for transient gene expression analysis. Nat Protoc. 2007;2(7):1565–72. Epub 2007/06/23. doi: 10.1038/nprot.2007.199 . 17585298

[22] Frearson EM, Power JB, Cocking EC. The isolation, culture and regeneration of Petunia leaf protoplasts. Dev Biol. 1973;33(1):130–7. Epub 1973/07/01. doi: 10.1016/0012-1606(73)90169-3 . 4789596

[23] Menczel L, Nagy F, Kiss ZR, Maliga P. Streptomycin resistant and sensitive somatic hybrids of Nicotiana tabacum + Nicotiana knightiana: correlation of resistance to N. tabacum plastids. Theor Appl Genet. 1981;59(3):191–5. Epub 1981/03/01. doi: 10.1007/BF00264975 . 24276446

[24] Mlotshwa Sizolwenkosi, Yang Zhiyong, Kim Yunju, Chen Xuemei. Floral patterning defects induced by Arabidopsis APETALA2 and microRNA172 expression in Nicotiana benthamiana. Plant Mol Biol. 2006; 61(4–5):781–93. doi: 10.1007/s11103-006-0049-0 16897492 PMC3574581

[25] Choi SH, Lee MH, Jin DM, Ju SJ, Ahn WS, Jie EY, et al. TSA Promotes CRISPR/Cas9 Editing Efficiency and Expression of Cell Division-Related Genes from Plant Protoplasts. Int J Mol Sci. 2021;22(15). Epub 2021/08/08. doi: 10.3390/ijms22157817 ; PubMed Central PMCID: PMC8346083. 34360584 PMC8346083

[26] Mengel A, Ageeva A, Georgii E, Bernhardt J, Wu K, Durner J, et al. Nitric Oxide Modulates Histone Acetylation at Stress Genes by Inhibition of Histone Deacetylases. Plant Physiol. 2017;173(2):1434–52. Epub 2016/12/17. doi: 10.1104/pp.16.01734 ; PubMed Central PMCID: PMC5291017. 27980017 PMC5291017

[27] Xu M, Du Q, Tian C, Wang Y, Jiao Y. Stochastic gene expression drives mesophyll protoplast regeneration. Sci Adv. 2021;7(33). Epub 2021/08/13. doi: 10.1126/sciadv.abg8466 ; PubMed Central PMCID: PMC8357238. 34380624 PMC8357238

[28] Lardon R, Wijnker E, Keurentjes J, Geelen D. The genetic framework of shoot regeneration in Arabidopsis comprises master regulators and conditional fine-tuning factors. Commun Biol. 2020;3(1):549. Epub 2020/10/04. doi: 10.1038/s42003-020-01274-9 ; PubMed Central PMCID: PMC7532540. 33009513 PMC7532540

[29] Zhang TQ, Lian H, Zhou CM, Xu L, Jiao Y, Wang JW. A Two-Step Model for de Novo Activation of WUSCHEL during Plant Shoot Regeneration. Plant Cell. 2017;29(5):1073–87. Epub 2017/04/09. doi: 10.1105/tpc.16.00863 ; PubMed Central PMCID: PMC5466026. 28389585 PMC5466026

[30] Yang K, Zhu L, Wang H, Jiang M, Xiao C, Hu X, et al. A conserved but plant-specific CDK-mediated regulation of DNA replication protein A2 in the precise control of stomatal terminal division. Proc Natl Acad Sci U S A. 2019;116(36):18126–31. Epub 2019/08/23. doi: 10.1073/pnas.1819345116 ; PubMed Central PMCID: PMC6731640. 31431532 PMC6731640

[31] Boruc J, Van den Daele H, Hollunder J, Rombauts S, Mylle E, Hilson P, et al. Functional modules in the Arabidopsis core cell cycle binary protein-protein interaction network. Plant Cell. 2010;22(4):1264–80. Epub 2010/04/22. doi: 10.1105/tpc.109.073635 ; PubMed Central PMCID: PMC2879739. 20407024 PMC2879739

[32] Chen K, Wang Y, Zhang R, Zhang H, Gao C. CRISPR/Cas Genome Editing and Precision Plant Breeding in Agriculture. Annu Rev Plant Biol. 2019;70:667–97. Epub 2019/03/06. doi: 10.1146/annurev-arplant-050718-100049 . 30835493

[33] Jacquier NMA, Gilles LM, Pyott DE, Martinant JP, Rogowsky PM, Widiez T. Puzzling out plant reproduction by haploid induction for innovations in plant breeding. Nat Plants. 2020;6(6):610–9. Epub 2020/06/10. doi: 10.1038/s41477-020-0664-9 . 32514145

[34] Zhang N, Laux T. Epigenetically jump starting de novo shoot regeneration. EMBO J. 2018;37(20). Epub 2018/10/03. doi: 10.15252/embj.2018100596 ; PubMed Central PMCID: PMC6187219. 30275268 PMC6187219

[35] Kumar V, Van Staden J. New insights into plant somatic embryogenesis: an epigenetic view. Acta Physiologiae Plantarum. 2017;39(9):194. doi: 10.1007/s11738-017-2487-5

[36] Turner BM. Histone acetylation and an epigenetic code. Bioessays. 2000;22(9):836–45. Epub 2000/08/17. doi: 10.1002/1521-1878(200009)22:9&lt;836::AID-BIES9&gt;3.0.CO;2-X . 10944586

[37] Feng W, Michaels SD. Accessing the Inaccessible: The Organization, Transcription, Replication, and Repair of Heterochromatin in Plants. Annu Rev Genet. 2015;49:439–59. Epub 2015/12/04. doi: 10.1146/annurev-genet-112414-055048 . 26631514

[38] Ma X, Zhang C, Zhang B, Yang C, Li S. Identification of genes regulated by histone acetylation during root development in Populus trichocarpa. BMC Genomics. 2016;17:96. Epub 2016/02/06. doi: 10.1186/s12864-016-2407-x ; PubMed Central PMCID: PMC4743431. 26847576 PMC4743431

[39] Martinez O, Arjones V, Gonzalez MV, Rey M. Histone Deacetylase Inhibitors Increase the Embryogenic Potential and Alter the Expression of Embryogenesis-Related and HDAC-Encoding Genes in Grapevine (Vitis vinifera L., cv. Mencia). Plants (Basel). 2021;10(6). Epub 2021/07/03. doi: 10.3390/plants10061164 ; PubMed Central PMCID: PMC8228518. 34201224 PMC8228518

[40] Tanaka M, Kikuchi A, Kamada H. The Arabidopsis histone deacetylases HDA6 and HDA19 contribute to the repression of embryonic properties after germination. Plant Physiol. 2008;146(1):149–61. Epub 2007/11/21. doi: 10.1104/pp.107.111674 ; PubMed Central PMCID: PMC2230551. 18024558 PMC2230551

[41] Liu C, Li LC, Chen WQ, Chen X, Xu ZH, Bai SN. HDA18 affects cell fate in Arabidopsis root epidermis via histone acetylation at four kinase genes. Plant Cell. 2013;25(1):257–69. Epub 2013/01/31. doi: 10.1105/tpc.112.107045 ; PubMed Central PMCID: PMC3584540. 23362208 PMC3584540

[42] Manghwar H, Lindsey K, Zhang X, Jin S. CRISPR/Cas System: Recent Advances and Future Prospects for Genome Editing. Trends Plant Sci. 2019;24(12):1102–25. Epub 2019/11/16. doi: 10.1016/j.tplants.2019.09.006 . 31727474

