# Supplementary material for: Effects of TSA, NaB, Aza in Lactuca sativa L. protoplasts and effect of TSA in Nicotiana benthamiana protoplasts on cell division and callus formation
Source: PLoS One. 2023 Feb 24;18(2):e0279627. doi: 10.1371/journal.pone.0279627 (PMC9956655; doi:10.1371/journal.pone.0279627)
Supplement: S1 Raw images — Total protein extracts were obtained from tobacco protoplasts after 6 h of TSA treatments. The level of H3 histone and H3 histone acetylation were determined via western blot analysis using anti-H3 and anti-AcH3 antibodies. (PDF) [file pone.0279627.s001.pdf]

S1 raw images. Raw western blot data using anti-H3 and anti-AcH3 antibodies in Fig. 8. Total protein extracts were obtained from tobacco protoplasts after 6 h of TSA treatments. The level of H3 histone and H3 histone acetylation were determined via western blot analysis using anti-H3 and anti-AcH3 antibodies.

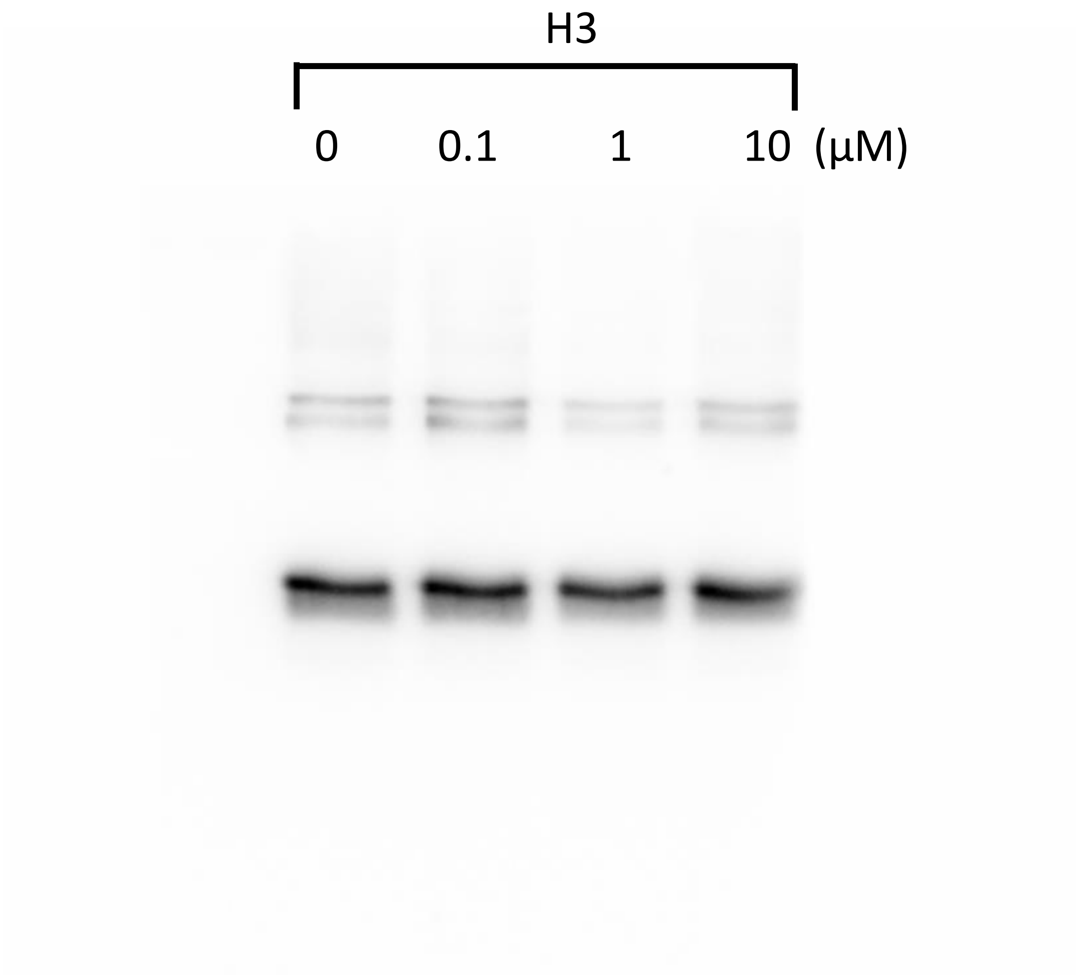

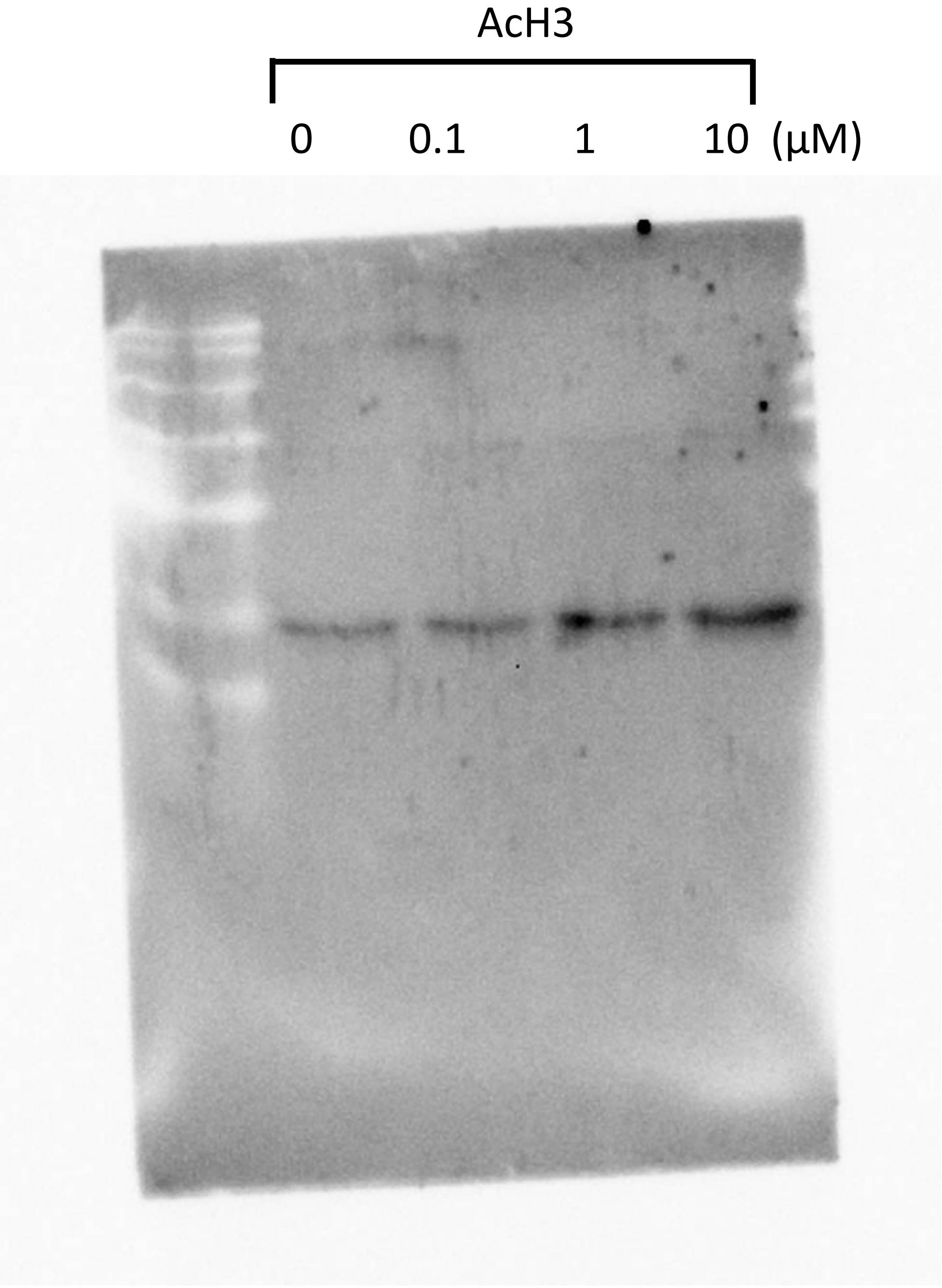
AcH3
0
0.1
1
10
(μM)
