# Supplementary material for: Effects of TSA, NaB, Aza in Lactuca sativa L. protoplasts and effect of TSA in Nicotiana benthamiana protoplasts on cell division and callus formation
Source: PLoS One. 2023 Feb 24;18(2):e0279627. doi: 10.1371/journal.pone.0279627 (PMC9956655; doi:10.1371/journal.pone.0279627)
Supplement: S1 Fig — A Freshly isolated mesophyll protoplasts cultured on B56I medium without TSA. B After 7 days of culture on B56I medium without TSA. C Freshly isolated mesophyll protoplasts cultured on B56I medium with 1 uM TSA. D After 7 days of culture on B56I medium with 1 uM TSA. Cell budding was observed after 7 days of culture. Scale bars represent 50 μm. (DOCX) [file pone.0279627.s002.docx]

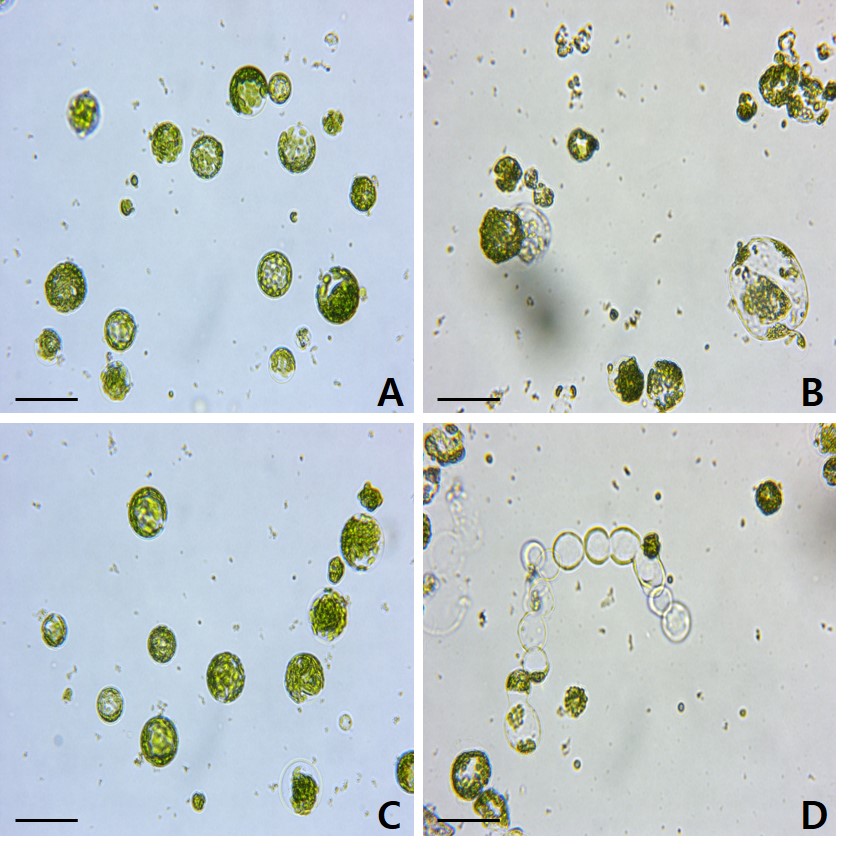


**S2 Fig. Effect of TSA on cell budding from mesophyll protoplasts of *N. benthamiana*. A** Freshly isolated mesophyll protoplasts cultured on B56I medium without TSA. **B** After 7 days of culture on B56I medium without TSA. **C** Freshly isolated mesophyll protoplasts cultured on B56I medium with 1 uM TSA. **D** After 7 days of culture on B56I medium with 1 uM TSA. Cell budding was observed after 7 days of culture. Scale bars represent 50 μm.
