# Supplementary material for: Effects of TSA, NaB, Aza in Lactuca sativa L. protoplasts and effect of TSA in Nicotiana benthamiana protoplasts on cell division and callus formation
Source: PLoS One. 2023 Feb 24;18(2):e0279627. doi: 10.1371/journal.pone.0279627 (PMC9956655; doi:10.1371/journal.pone.0279627)
Supplement: S1 Table — (DOCX) [file pone.0279627.s003.docx]

**S1 Table. Effect of TSA on cell budding from mesophyll protoplasts of *N. benthamiana* after 7 days of culture.**

| Culture medium | TSA treatment  (1 μM) | Frequency of cell division | Frequency of cell budding^1^ |
| --- | --- | --- | --- |
| B56I | - | 0 | 5.0 ± 1.7 |
| B56I | + | 0 | 12.0 ± 2.6 |

^1^ : The frequency of cell division and budding from protoplasts was determined by counting the number of cells with division and budding.
